# Intestinal ischemia secondary to superior mesenteric venous thrombosis—A case report

**DOI:** 10.1016/j.ijscr.2018.10.039

**Published:** 2018-10-29

**Authors:** Jason Cui, Brian Kirkby

**Affiliations:** General Surgical Department, Caboolture Hospital, Caboolture, QLD 4510, Australia

**Keywords:** Mesenteric vein thrombosis, Inflammatory bowel disease, Intestinal ischemia, Conservative management, Case report, General surgery

## Abstract

Introduction: Acute mesenteric venous thrombosis is a rare but potentially fatal condition with superior mesenteric vein being the most common site of thrombosis development [1]. It is more common in patients with underlying disorders which cause disruptions to Virchow’s Triad of hypercoagulability, stasis and endothelial injury. The disease is often associated with intestinal ischemia in its acute form, further complicating its management.

Presentation of case: We present a case of acute superior mesenteric venous thrombosis resulting in jejunal ischemia in a 60-year-old Caucasian male with possible ulcerative colitis. A computed tomography (CT) scan demonstrated features of intestinal infarction. However, the patient was stable after initial resuscitation with a non-peritonitic abdomen and was subsequently managed with conservative measures.

Discussion: We have illustrated a case of a patient with acute mesenteric venous thrombosis with potential bowel compromise. Despite concerning CT features, the patient was clinically stable and responded to conservative management. We hope to emphasise that although radiology plays a crucial role in modern medicine, it is important to make therapeutic decisions based on clinical findings.

Conclusion: Acute mesenteric venous thrombosis is a complex entity. It requires a multidisciplinary team approach to plan for the most appropriate treatment strategy suitable for each patient as all options are associated with significant risks. The underlying cause should be established as this will determine any long-term management necessary to prevent recurrence.

## Highlights

• Mesenteric venous thrombosis (MVT) can be fatal with the superior mesenteric vein being the most common site of thrombus formation.
• Intestinal ischemia often accompanies MVT in the acute setting, complicating its management.
• Patients who present with MVT should be screened for conditions that cause disruption to Virchow’s Triad.

## 1 Introduction

This case report is in line with the SCARE criteria [2].

Acute mesenteric venous thrombosis is a rare but potentially fatal condition with a mortality rate of 20–30% [3]. Like all thrombotic events, the formation of mesenteric venous thrombosis is a result of Virchow’s Triad (Endothelial injury, stasis of flow and hypercoagulability). However, the specific aetiology may not be obvious. Although it is relatively easy to diagnose on imaging, the condition poses a therapeutic dilemma as both conservative and operative means are associated with significant risks.

## 2 Case report

A 60-year-old Caucasian male patient was brought to the resuscitation bay of our Emergency Department after a syncopal episode and was noted to be in shock. His initial observations include Heart Rate 126/min, Blood Pressure 102/79 mmHg, Respiratory rate 32/min, temperature 36.8C and oxygen saturation of 98% on 2 L of nasal prongs. The patient had a Glasgow Coma Scale of 14 and was clinically dehydrated. Abdominal examination revealed a distended abdomen that was tender in the left upper quadrant with no evidence of peritonism. Laboratory tests showed polycythaemia with Haemoglobin of 189 g/L and white cell count of 20.3 × 10^9^/L with preserved renal and hepatic functions. Arterial blood gas analysis was consistent with normal anion gap metabolic acidosis with a pH of 7.28 and lactate of 4.5. The patient responded to initial fluid resuscitation.

Further history was obtained: he had a 2-day history of progressively worsening upper abdominal pain associated with multiple episodes of dark coloured emesis and loose bowel motions. The pain was exacerbated by oral intake. He had a background of ulcerative colitis diagnosed at the age of 40 which required one course of oral steroid with no further follow up or treatment.

**Fig. 1:**
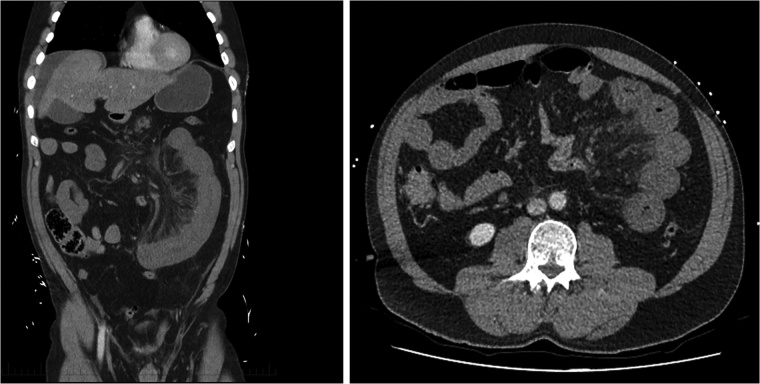
CT demonstrating intestinal ischemia involving a long segment of the jejunum with evidence of bowel wall thickening and fat stranding.

**Fig. 2:**
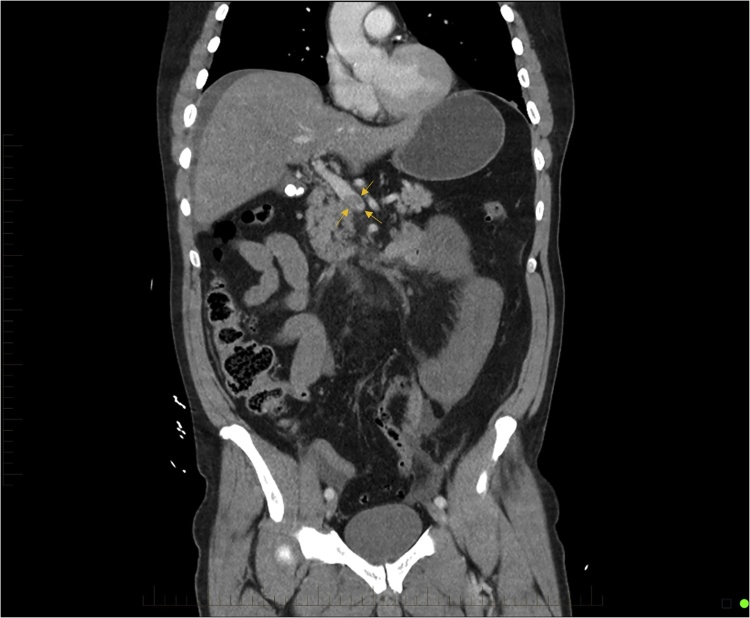
Superior mesenteric venous thrombosis with extension into the portal vein confluence (Yellow Arrows).

As the patient remained in a stable state, a CT abdomen was performed which demonstrated a long segment of superior mesenteric vein (SMV) thrombus extending into the portal vein. This resulted in venous engorgement with associated thickening of jejunal wall and extensive mesenteric stranding suggestive of intestinal ischemia. There is also a moderate amount of free fluid with no evidence of intramural gas or perforation (Fig. 1, Fig. 2).

Acute mesenteric venous thrombosis was diagnosed and management options were explored. Although the CT appearances were sinister and suggestive of intestinal ischemia, the patient had no signs of peritonism. Thus, decision was made for conservative management. Heparin infusion was commenced, nasogastric tube was inserted and the patient was placed on bowel rest. Central venous line, arterial line and a urinary catheter were inserted and the patient was transferred to the intensive care unit (ICU).

Serial abdominal examinations were performed. Over the subsequent 24 h, the patient remained clinically stable and showed improvement in his metabolic acidosis. His abdominal examination was consistent with ileus with no peritoneal signs. Total parenteral nutrition was commenced in anticipation to delays in re-establishing enteral nutritional intake.

The patient was stepped down from ICU on day 6, his abdominal tenderness improved and showed signs of resolving ileus. Episodes of melaena were observed with no haemodynamic changes and with stable haemoglobin levels. Oral intake was slowly introduced and gradually upgraded. He was subsequently discharged on day 17 post admission after transitioning onto oral anticoagulation with warfarin.

The patient was followed up in the outpatient clinic one month after discharge. He had returned to his baseline function with no gastroenterological symptoms. Extensive investigations were performed to assess for underlying causes of the patient’s SMV thrombosis. These included thrombophilia and myelodysplasia screens, malignancy screens and an echocardiogram. Unfortunately, no specific causes were detected. His initial polycythaemia also resolved after adequate fluid replacement and was attributed to severe dehydration on presentation. A follow up colonoscopy was performed which showed no evidence of mucosal inflammation or features to suggest inflammatory bowel disease.

## 3 Discussion

The incidence of mesenteric venous thrombosis is 2.7 per 100,000 patient-years [4] and accounts for 5–15% of mesenteric ischemia [5]. Superior mesenteric vein is the most common site of thrombi formation, resulting in impaired venous return and subsequent venous engorgement and bowel ischemia [1]. The most common sites involved are ileum and jejunum [6]. The potential to develop transmural infarction is of concern, as the loss of bowel integrity can lead to bacterial translocation and potential fatal sequelae. Interestingly, mesenteric venous thrombosis with portal vein involvement has a lower risk of developing transmural infarction compared to isolated mesenteric vein alone [7].

Comparable to venous thrombosis in general, Virchow’s triad is crucial in the pathogenesis of mesenteric vein thrombosis. In this patient, extensive investigations have been performed to exclude underlying malignancies along with haematological and cardiac disorders. Intra-abdominal inflammatory processes are also important risk factors to consider. A study by Hatoum et al. in 2005 have analysed 545 patients with inflammatory bowel disease of which 6 (1.1%) have developed mesenteric venous thrombosis. In the 6 patients, all had underlying hypercoagulability, low flow state, uncontrolled inflammation or was post-operative [8]. The patient in this case had no evidence to suggest an acute exacerbation of his ulcerative colitis prior to his acute presentation with a follow up colonoscopy which showed no evidence of inflammatory bowel disease, therefore it is difficult to attribute ulcerative colitis to the formation of SMV thrombus.

Multiple options are available for the management of mesenteric venous thrombosis. In patients with peritoneal signs to suggestive bowel infarction or perforation or those who failed to progress with conservative management, operative intervention may be necessary. Other options include anticoagulation therapy, local or systemic thrombolysis, interventional or surgical thrombectomy. Guglielmi et al in 2005 has reported on a similar case of a 40-year-old female patient with mesenteric and portal vein thrombosis and active ulcerative colitis who was managed successfully with local thrombolysis. Alteplase was administered via transhepatic route with a follow up angiogram at 48 h showing complete resolution of the thrombus [10]. The study suggested potential therapeutic benefits over surgical intervention in terms of mortality as well as benefits compared to systemic anticoagulation. However, there are no high-level evidence to compare the management options of mesenteric venous thrombosis currently.

Clinically, the patient in this case was unwell with a distended, tender abdomen. He was severely hypovolaemic with metabolic acidosis and high lactate. The findings on CT were also highly suspicious for compromised bowel. However, he has responded to initial resuscitation with no appreciable peritonism. An operation would be associated with extremely high risks. Bowel resection may be inevitable and further ischemia may develop post-operatively leading to potentially fatal outcomes. We have consulted the vascular and interventional radiology teams to explore options of thrombolysis and percutaneous thrombectomy, both of which have been deemed inappropriate as there is little evidence with significant risks. After careful assessment of benefits and risks in conjunction with extensive family discussion, initial conservative management with anticoagulation therapy was attempted with scrupulous assessment of the patient’s progress.

The prognosis of patients with mesenteric venous thrombosis who responded to conservative management has been reported to be as high as 93% at 3 years [9]. However, it is often limited by the severity of the underlying disease. Long term complications such as recurrence of thrombosis and intestinal ischemic strictures can also develop with variable reported incidences in small studies. Given the unclear aetiology and the potential fatal sequalae of thrombus recurrence in this patient, lifelong anticoagulation was recommended.

## Conflicts of interest

There are no conflicts of interest, financial, personal or otherwise which could influence bias.

## Funding source

No funding was needed for this case report, except for the amount required if published.

## Ethical approval

Ethical approval was not required for this case report.

## Consent

Consent was obtained from the patient to write this case report. The accompanying images. identifying details have been omitted.

## Author contribution

Dr Jason Cui – Corresponding author. Review of patient, drafting of article and approval of final submission.

Dr Brian Kirkby – Surgeon responsible for the management of the patient, review and approval of final submission.

## Registration of research studies

NA.

## Guarantor

Dr Jason Cui – General Surgical Department, Caboolture Hospital, Caboolture, QLD, 4510, Australia.

Dr Brian Kirkby – Director of Surgery, Caboolture Hospital, Mckean street, Caboolture, 4510, Australia.

## Provenance and peer review

Not commissioned, externally peer reviewed.
